# SERS Probing of Proteins in Gold Nanoparticle Agglomerates

**DOI:** 10.3389/fchem.2019.00030

**Published:** 2019-01-31

**Authors:** Gergo Peter Szekeres, Janina Kneipp

**Affiliations:** ^1^Department of Chemistry, Humboldt-Universität zu Berlin, Berlin, Germany; ^2^School of Analytical Sciences Adlershof, Berlin, Germany

**Keywords:** gold nanoparticles, surface-enhanced Raman scattering (SERS), protein corona, bovine serum albumin (BSA), DNA

## Abstract

The collection of surface-enhanced Raman scattering (SERS) spectra of proteins and other biomolecules in complex biological samples such as animal cells has been achieved with gold nanoparticles that are introduced to the sample. As a model for such a situation, SERS spectra were measured in protein solutions using gold nanoparticles in the absence of aggregating agents, allowing for the free formation of a protein corona. The SERS spectra indicate a varied interaction of the protein molecule with the gold nanoparticles, depending on protein concentration. The concentration-dependent optical properties of the formed agglomerates result in strong variation in SERS enhancement. At protein concentrations that correspond to those inside cells, SERS signals are found to be very low. The results suggest that in living cells the successful collection of SERS spectra must be due to the positioning of the aggregates rather than the crowded biomolecular environment inside the cells. Experiments with DNA suggest the suitability of the applied sample preparation approach for an improved understanding of SERS nanoprobes and nanoparticle-biomolecule interactions in general.

## Introduction

Surface-enhanced Raman scattering (SERS) using plasmonic metal nanoparticles has been applied to characterize complex biological samples, ranging from biomacromolecules such as nucleic acids (Kneipp and Flemming, 1986; Barhoumi and Halas, 2010) and proteins (Han et al., 2009; Blum et al., 2012; Bonifacio et al., 2014; Fazio et al., 2016) to living eukaryotic and prokaryotic cells (Kneipp et al., 2006; Ivleva et al., 2010; Aioub and El-Sayed, 2016; Fasolato et al., 2016) and tissues in whole animals (Charan et al., 2011; Kneipp et al., 2012) for several decades now. The sensitivity of the SERS signal with respect to interactions at the surface of the plasmonic nanoparticles makes it specifically useful for studies of molecular contacts of nanoparticles in biological systems, that is, in-between and within cells of animal, plant, or bacterial origin.

Obtaining SERS data from solutions of biomolecules, specifically those of proteins, is challenging, and often aggregates of nanostructures must be generated due to the high local fields (Blatchford et al., 1982; McMahon et al., 2011; Stockman, 2011) that can then be exploited for SERS. As examples, the aggregation of silver nanoparticles allows for the SERS detection of some proteins (Reymond-Laruinaz et al., 2016) at low concentrations in solution (Xu et al., 1999; Han et al., 2009), or re-structuring of nanostructured silver films induced by the protein molecules themselves yielded SERS spectra at surface concentrations on the order of fM per mm^2^ (Drachev et al., 2005). The immobilization of SERS-active nanostructures, together with the analyte biomolecule, on flat surfaces (Iosin et al., 2011; Brulé et al., 2016), or by optical tweezing (Fazio et al., 2016), or the application of specially tailored plasmonic substrates (Alvarez-Puebla et al., 2011) can be used to conduct efficient SERS experiments with protein solutions.

Nevertheless, these situations are different from probing nanoparticle-biosystem interfaces without protein (or biomolecule) purification. Specifically, the delivery of plasmonic nanostructures into a biological system, such as a live cell, requires the use of individual, non-aggregated nanostructures, which are known to provide very low SERS enhancement (Wang and Kerker, 1981; Joseph et al., 2011), but which are usually later processed by their biomolecular environment and form aggregates that yield high SERS enhancement (Buchner et al., 2014). Despite the fact that also intracellular SERS spectra are dominated by contributions from protein molecules (Buchner et al., 2014), comparing SERS spectra of proteins in solution with those obtained directly from a cell is challenging. In particular, the protein concentration is higher in cells than in dilute solutions (Brown, 1991; Milo, 2013), and although proteins facilitate the formation of nanoparticle aggregates both in cells and *in vitro* (Zhang et al., 2009; Bharti et al., 2011; Moerz et al., 2015; Fazio et al., 2016) where they move around freely due to Brownian motion (Jia et al., 2007), additional positioning of nanoaggregates formed by cells was found to occur (Ando et al., 2011; Drescher et al., 2013b).

Here, we discuss probing of proteins by SERS in solution using gold nanoparticles in the absence of immobilizing agents or aggregating salts so that neither the tertiary structure of the proteins nor the surface properties of the nanoparticles are affected. We prepared samples of biomolecule solutions with gold nanoparticles varying in concentration by seven orders of magnitude. This preparation allows for the free protein corona formation, in analogy to the situation in cells and culture media. The collected SERS spectra indicate a strong variation in enhancement and spectral information, both being the result of a varying interaction of the same protein molecule with the nanoparticles with altered protein concentration. The results enable a discussion about the possible formation of gold nanoaggregates with high SERS enhancement in very crowded molecular environments such as the compartments of animal cells.

## Materials and Methods

### Preparation of Gold Nanoparticles

Milli-Q water was used in all experiments. Gold(III) chloride trihydrate (Sigma-Aldrich, Steinheim, Germany) and trisodium citrate dihydrate (Chemsolute, Renningen, Germany) were used for gold nanoparticle synthesis by the recipe described in Lee and Meisel (1982). The nominal nanoparticle concentration was 0.4 nM, based on the diameter distribution of 155 gold nanoparticles (32 ± 7 nm) and their final pH was 4.54. The 32 nm average size and the 0.4 nM nominal stock concentration of gold nanoparticles has been shown to be adequate for the SERS investigation of biologically relevant molecules and living cells as well (Kneipp et al., 2006; Drescher et al., 2013a).

### Raman Experiments

Raman and SERS spectra were measured with a microspectroscopic setup containing a single-stage spectrograph equipped with a liquid-nitrogen-cooled charge-coupled device detector. Excitation of the Raman scattering was done in 180° backscattering geometry through a 60x immersion objective (Olympus, Germany) using a wavelength of 785 nm and a laser intensity of 5.7 × 10^5^ W/cm^2^.

Spectra were collected from the ~10 fL focal volume created in droplets of 50 μL sample solution with or without gold nanoparticles on CaF_2_ slides. Acquisition time per spectrum varied between 1 and 10 s depending on the sample.

### Protein Samples

The protein concentration was chosen to be in the range of intracellular protein concentration values (based on data from previous work, this range corresponds to a concentration interval of 0.02–0.55 g/mL for BSA). Milo (2013) 0.5 g of BSA (Sigma-Aldrich, Germany) were dissolved in 1 mL Milli-Q water, then 50 μL of this solution were transferred to a calcium fluoride plate, and Raman spectra were collected from the droplet with an acquisition time of 10 s.

For SERS measurements, mixtures of gold nanoparticles and BSA were prepared at different gold nanoparticle: BSA molar ratios. The final nominal concentration of gold nanoparticles in the SERS experiments was estimated to be in the range of 0.3–0.4 nM.

In the first experiment, SERS spectra were collected from a sample with a gold nanoparticle: BSA ratio of 1:10^7^ corresponding to a 3-fold dilution of the initial solution of 0.5 g/mL, by mixing 50 μL 0.5 g/mL protein solution with 100 μL gold nanoparticles, yielding a final protein concentration in the range of intracellular protein concentration (0.15 g/mL) (Milo, 2013). The SERS spectra were collected immediately after sample preparation with an acquisition time of 5 s.

In the second experiment, a sample with a gold nanoparticle: BSA molar ratio of 1:40,000 was prepared by adding 5 μL of 0.01 g/mL BSA solution to 50 μL of gold nanoparticles (final BSA concentration of ~15 μM). In the light microscope, micrometer-sized aggregates of nanoparticles and protein were observed. Focusing on the bottom of the droplet, where the precipitated aggregates had settled, SERS spectra were collected with an acquisition time of 1 s immediately after sample preparation.

In a third experiment, the 0.01 g/mL BSA solution was diluted 10^5^-fold, and 5 μL of this BSA solution was added to 50 μL gold nanoparticles to obtain samples with a protein concentration of ~150 pM, corresponding to a molar gold nanoparticle: BSA ratio of 2.5:1. The samples were incubated for 90 min before SERS spectra were measured focusing on the settled aggregates, with an acquisition time of 1 s.

### DNA Samples

3T3 cells (DSMZ, Braunschweig, Germany) were cultured at 37°C in humid air with 5% CO_2_ in Dulbecco's Modified Eagle Medium (Biochrom, Berlin, Germany) with 10% Fetal Calf Serum (Biochrom, Berlin, Germany) and 1% of ZellShield^TM^ (Biochrom, Berlin, Germany) as described in Buchner et al. (2014). For DNA extraction, the culture medium was removed from a cell flask containing ca. 10^6^ 3T3 cells, and the cells were washed by phosphate buffer solution (Biochrom, Berlin, Germany). After detaching the cells from the wall of the flask by trypsin-EDTA solution (Biochrom, Berlin, Germany), subsequent centrifugation resulted in a cell pellet (every centrifugation step was performed at 14,000 rpm). This pellet was resuspended in 100 μL water, and full cell lysis was performed by repeating the cycle of freezing in liquid nitrogen and thawing at 37°C in thermostated water four times. The sample was centrifuged, the supernatant was transferred to a new Eppendorf tube, and the sediment made up by cell debris was discarded. Ice-cold ethanol (70%, VWR Chemicals, Fontenay-sous-Bois, France) was added to the supernatant for the precipitation of DNA, and the sample was then centrifuged. After discarding the supernatant, the pellet–consisting of DNA–was resuspended in pure ethanol and centrifuged once more to make sure that all the proteins are removed. Following the centrifugation the supernatant was discarded, the DNA pellet was dried, then dissolved in 1 mL Milli-Q water. Five μL of this solution was added to 50 μL of gold nanoparticles, which is consistent with SERS experiments on protein solutions.

### UV-Vis Absorbance Spectra

Gold nanoparticles and the sample containing a BSA solution-nanoparticle mixture with a molar gold nanoparticle: BSA ratio of 1:40,000, and of 2.5:1, respectively, were measured in a quartz cuvette, and spectra were acquired with a Jasco V-670 spectrometer in the wavelength range of 300–1,000 nm.

### Brownian Motion of the Gold Nanoparticles in the Protein Solutions

The displacement of the nanoparticles in a solution can be described as

(1) $$\begin{array}{l} <r^{2}>\;=\;\frac{2k_{B}T}{3\pi \eta \alpha }t, \end{array}$$

where < *r*^2^ > is the time-averaged 2D displacement of the particle, *k*_*B*_ is the Boltzmann constant, *T* is the temperature, *t* is the time, η is the dynamic viscosity, and α is the particle radius (Jia et al., 2007). The viscosity of BSA solutions changes exponentially with the concentration:

(2) $$\begin{array}{l} {\eta =\eta }_{0}\;e^{\frac{\left[\eta \right]c}{1-\frac{k}{\nu }\left[\eta \right]c}}, \end{array}$$

where η is the dynamic viscosity, η_0_ is the viscosity of the solvent, *[*η*]* is the intrinsic viscosity in mL/g, *k* is the self-crowding factor, ν is the Simha shape parameter, and *c* is the BSA concentration in g/mL (Yadav et al., 2011). We estimated the viscosity of the SERS sample for the different gold nanoparticle: BSA ratios by substituting η_0_ with 0.8 cP (approximate viscosity of the nanoparticle colloid; Abdelhalim et al., 2011), *[*η*]* with 3.8 mL/g (at pH = 4.54) (Tanford and Buzzell, 1956), and the *k/*ν ratio with 0.45, as determined by Yadav et al. (2011).

## Results and Discussion

For normal Raman and SERS experiments, BSA solutions with different concentrations were prepared. The normal Raman experiments were performed on a sample with 0.5 g/mL BSA concentration that corresponds to that of the crowded environment inside a cell (Milo, 2013). In the SERS experiments, the same amount of citrate stabilized gold nanoparticles (corresponding to a final nominal gold nanoparticle concentration of 0.3–0.4 nM) was added to BSA solutions with different concentrations to reach final gold nanoparticle: BSA molar ratios of 1:10^7^, 1:40,000, and 2.5:1, respectively. To obtain the ratio of 1:10^7^, the original BSA solution was diluted 3-fold, maintaining the similarity to the protein concentration in a cell.

**Figure 1:**
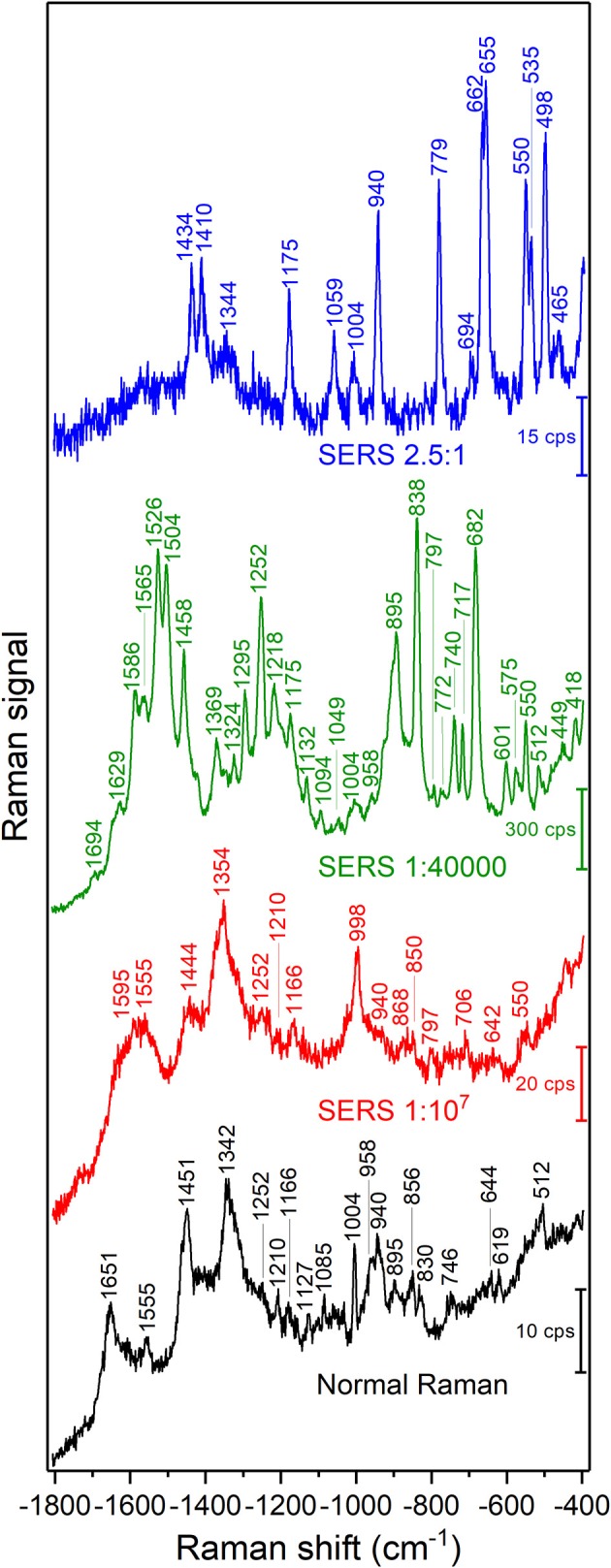
SERS spectra of BSA with gold nanoparticles with different gold nanoparticle: BSA molar ratios as indicated in the graph. Excitation wavelength: 785 nm, excitation intensity 5.7 × 10^5^ W/cm^2^, acquisition time per spectrum: 5 s for normal Raman, 1 s for SERS spectra.

A representative Raman spectrum of BSA in solution and SERS spectra of the different samples are shown in Figure 1. The normal Raman spectrum (Figure 1, bottom) and the SERS spectrum of the sample with the gold nanoparticle: BSA molar ratio of 1:10^7^ are very similar both in band positions (Tables 1, 2) and signal-to-noise ratio. The normal Raman spectrum is in good accordance with previous data (Lin and Koenig, 1976). The spectrum of the sample with the gold nanoparticle: BSA ratio of 1:10^7^ (Figure 1, red trace) shows some altered band positions and very small enhancement of up to a factor of ~6 for some of the bands, e.g., the phenylalanine ring breathing mode ~1,004 cm^−1^, and indicates that most of the signal is a normal Raman signal. This is in agreement with the fact that only a very small fraction of BSA molecules in the focal volume can interact with the nanoparticles (~100 BSA molecules/nanoparticle) (Dominguez-Medina et al., 2012), and that the absolute protein concentration of 0.15 g/mL is high enough to yield a normal Raman signal. The enhancement of around one order of magnitude indicates that those nanoparticles that contribute to the SERS signal do so by acting as individual nanostructures, and cannot form aggregates (Joseph et al., 2011).

**Table 1:** Raman shift values and tentative band assignments of the normal Raman spectrum of BSA in solution.

**Raman shift (cm^**−1**^)**	**Tentative assignment**	**References**
1,651	Amide I	Lin and Koenig, 1976
1,555	Trp	Lin and Koenig, 1976
1,451	δ(C-H_2_/CH_3_)	Lin and Koenig, 1976
1,336	δ(C-H)	Lin and Koenig, 1976
1,210	Tyr, Phe	Lin and Koenig, 1976
1,127	C-N	Lin and Koenig, 1976
1,085	C-N	Lin and Koenig, 1976
1,004	R breath	Lin and Koenig, 1976
958	Trp, Val	Lin and Koenig, 1976; Rygula et al., 2013
940	δ(C-C-N)_symm_, α-helical skeletal	Lin and Koenig, 1976
895	Trp	Lin and Koenig, 1976; Rygula et al., 2013
856	Tyr	Lin and Koenig, 1976
830	Tyr	Lin and Koenig, 1976
746	Trp, ν(C-S)	Lin and Koenig, 1976; Hornemann et al., 2013; Rygula et al., 2013
644	Tyr, ν(C-S)	Lin and Koenig, 1976; Peticolas, 1995; Rygula et al., 2013
619	Tyr, ν(C-S)	Lin and Koenig, 1976
512	ν(S-S)	Lin and Koenig, 1976

*δ, deformation; breath, breathing; ν, stretching; symm, symmetric*.

**Table 2:** Raman shift values and tentative band assignments of the SERS spectra of BSA.

**Raman shift (cm^**−1**^)**	**Tentative assignment**	**References**
1,694	Amide I	Rygula et al., 2013
1,629	Amino acids	Rygula et al., 2013; Madzharova et al., 2017
**1,595**	Phe, Tyr: ν(R), ν (COO^−^)asymm, OH mode	Drescher et al., 2013a; Hornemann et al., 2013; Madzharova et al., 2017
1,586	δ(R), ν(R)	Hornemann et al., 2013; Madzharova et al., 2017
1,577	Trp, NH_2_ sciss, ν(R,r)	Drescher et al., 2013a; Madzharova et al., 2017
1,565	Amide II	Hornemann et al., 2013; Rygula et al., 2013
**1,555**	Trp: ν(R), ν(r), amide II	Drescher et al., 2013a; Madzharova et al., 2017
1,526	$\delta (\text{N}{\text{H}}_{3}^{+}$)Lys, amide II	Peticolas, 1995; Hornemann et al., 2013
1,504	Amide II, ν(R,r) Trp, ν(C-H)	Hornemann et al., 2013; Fazio et al., 2016; Madzharova et al., 2017
**1,458**	δ(C-H), δ(CH_2_/CH_3_)	Lin and Koenig, 1976; Drescher et al., 2013a; Rygula et al., 2013
1,444	δ(C-H) of CH_2_	Rygula et al., 2013
1,434	δ(CH_2_)	Zhu et al., 2011
**1,425**	δ(CH_2_)Cys	Lin and Koenig, 1976; Susi et al., 1983
**1,410**	δ(CH_3_), ν(COO^−^)	Lin and Koenig, 1976; Hornemann et al., 2013; Madzharova et al., 2017
1369	δ(CH_3_)symm	Drescher et al., 2013a
1354	γ(CH_2_)	Hornemann et al., 2013
1,344	γ(CH_2_,CH_3_), Trp(Cα-H)	Drescher et al., 2013a; Hornemann et al., 2013; Rygula et al., 2013
**1,324**	δ(C-H), δ(CH_2_/CH_3_)	Lin and Koenig, 1976; Drescher et al., 2013a; Hornemann et al., 2013
1,295	Amide III, C-H, C-C	Rygula et al., 2013; Madzharova et al., 2017
**1,252**	Amide III	Lin and Koenig, 1976; Hornemann et al., 2013; Rygula et al., 2013; Fazio et al., 2016
1,210; 1,218	Tyr, Phe, ν(C-C)	Hornemann et al., 2013; Madzharova et al., 2017
**1,175**	Tyr, ν(-C-N)	Lin and Koenig, 1976; Hornemann et al., 2013
**1,166**	Tyr	Lin and Koenig, 1976
**1,132**	ν(-C-N), Pro	Lin and Koenig, 1976; Hornemann et al., 2013; Rygula et al., 2013
**1,094**	ν(C-C),ν(C-N), H(r) bend	Lin and Koenig, 1976; Hornemann et al., 2013; Madzharova et al., 2017
**1,049;1,059**	ν(-C-N), Phe	Lin and Koenig, 1976; Hornemann et al., 2013; Rygula et al., 2013
**998;1,004**	R breathing	Lin and Koenig, 1976; Hornemann et al., 2013; Rygula et al., 2013; Fazio et al., 2016; Madzharova et al., 2017
**958**	ν(N−C_α_-C) skeletal	Lin and Koenig, 1976; Rygula et al., 2013
**940**	δ(C-C-N)symm, α-helical skeletal	Lin and Koenig, 1976)
**895**	Trp	Lin and Koenig, 1976; Rygula et al., 2013
868	Tyr	Fazio et al., 2016
850	Tyr: R breathing	Lin and Koenig, 1976; Madzharova et al., 2017
**838**	Tyr	Lin and Koenig, 1976; Peticolas, 1995; Hornemann et al., 2013; Rygula et al., 2013
**797**	ν(C-H), δ(-N-H)	Lin and Koenig, 1976; Hornemann et al., 2013
772;779	Trp, δ(-C-H)	Hornemann et al., 2013
**740**	Trp, ν(C-S)	Lin and Koenig, 1976; Peticolas, 1995; Rygula et al., 2013
**717**	ν(C-S), Trp	Lin and Koenig, 1976; Peticolas, 1995; Hornemann et al., 2013; Rygula et al., 2013
**706**	ν(C-S), δ(COO^−^)	Lin and Koenig, 1976; Peticolas, 1995; Rygula et al., 2013; Madzharova et al., 2017
694	C-C, C-O bend	Hernandez et al., 2010
682	δ(-C-H)	Hornemann et al., 2013
655;**662**	ν(C-S), Tyr	Lin and Koenig, 1976; Peticolas, 1995; Hornemann et al., 2013; Rygula et al., 2013
**642**	ν(C-S)	Lin and Koenig, 1976; Peticolas, 1995
601	δ(COO^-)	Zhu et al., 2011
575	ν(S-S)	Hornemann et al., 2013
550	ν(S-S)	Rygula et al., 2013; Fazio et al., 2016
535	δ(N-H), ν(S-S), δ(skeletal)	Peticolas, 1995; Drescher et al., 2013a; Hornemann et al., 2013; Rygula et al., 2013; Madzharova et al., 2017
**512**	ν(S-S)	Lin and Koenig, 1976; Peticolas, 1995; Rygula et al., 2013
**498**	ν(S-S)	Lin and Koenig, 1976; Drescher et al., 2013a; Hornemann et al., 2013; Rygula et al., 2013; Fazio et al., 2016
465	ν(C-S)	Hornemann et al., 2013
**449**	ν(C-S)	Lin and Koenig, 1976; Hornemann et al., 2013
**418**	Trp	Lin and Koenig, 1976; Hornemann et al., 2013

*sciss, scissoring; δ, deformation; ν, stretching; bend, bending; R, benzene ring; r, pyrrole ring. Bands appearing in the normal Raman spectrum of BSA solution are marked bold*.

When the protein concentration is decreased to a gold nanoparticle: BSA molar ratio of 1:40,000, while maintaining the concentration of gold nanoparticles, the formation of nanoparticle aggregates is observed in the Raman microscope. The signals in the spectrum increase drastically, and new bands appear. The vibrations at 838, 895, 1,252, 1,458, 1,504, and 1,526 cm^−1^, which are the same as in the normal Raman spectrum of BSA (marked in bold in Table 2) show very high relative intensities. Some, e.g., the vibration of tryptophan at 895 cm^−1^, known from the normal Raman spectrum (cf. Figure 1, bottom; Lin and Koenig, 1976) are absent from other SERS spectra (Hornemann et al., 2013; Fazio et al., 2016), including most of those shown here (Figure 1). Based on the assignments of the bands in the spectrum (Table 2), we conclude that an interaction of the protein with the nanoparticles' surface takes place in such a way that both the peptide backbone and the aromatic side chains of BSA are in the proximity of the nanoparticle surface: The signals of the aromatic side chains suggest the unfolding of the protein, since, as indicated by its crystallographic data (Figure 3A; Bujacz et al., 2014), these side chains are rarely present on the surface of BSA. The bands at 512, 550, and 575 cm^−1^, assigned to S-S stretching vibrations of disulfide bonds formed by cysteine, however, indicate that at least parts of the native structure remain intact. The bands at 1,252, 1,295, 1,504, 1,526, 1,565, 1,629, and 1,694 cm^−1^, which can be assigned to some components of the amide III, amide II, and the amide I bands, respectively, indicate the interaction of nanoparticles with the peptide backbone. This also points to a more intact, folded protein chain, where specific side chain-nanoparticle interactions are less likely to take place.

At ~150 pM (10^−8^ g/mL) BSA concentration, corresponding to a gold nanoparticle: BSA ratio of 2.5:1, the surface of the nanoparticles cannot be completely covered by BSA molecules. In this case, the aggregation, observed in the dark field of the Raman microscope by eye, must be based on bridging of individual nanoparticles by a protein molecule (Moerz et al., 2015). Comparing the SERS spectra (Figure 1, blue trace) to those of the other samples, in spite of a lower overall intensity, an increment in the relative intensities of the bands at 498, 550, 655, and 662 cm^−1^ is observed, all of which correspond to C-S and S-S vibrations. The crystal structure of BSA indicates that Cys and Met side chains are not exposed on the surface of the molecule (Figure 3B; Bujacz et al., 2014), therefore, the interaction of sulfuric residues with the gold nanoparticle surface would require unfolding of the native structure of BSA. Different from the sample with the gold nanoparticle: BSA ratio of 1:40,000 (Figure 1, green trace), no amide bands are present in this spectrum, which suggests a more unfolded structure, where specific side chain interactions become more preferred than peptide backbone interactions. This is in agreement with the expected aggregation process, since a protein molecule must undergo conformational changes while binding to two different nanoparticles at the same time. Nevertheless, while unfolding of the native structure is observed at this concentration, the presence of S-S vibrations, e.g., at 550 cm^−1^ indicates that the native folding is not completely disrupted.

**Figure 2:**
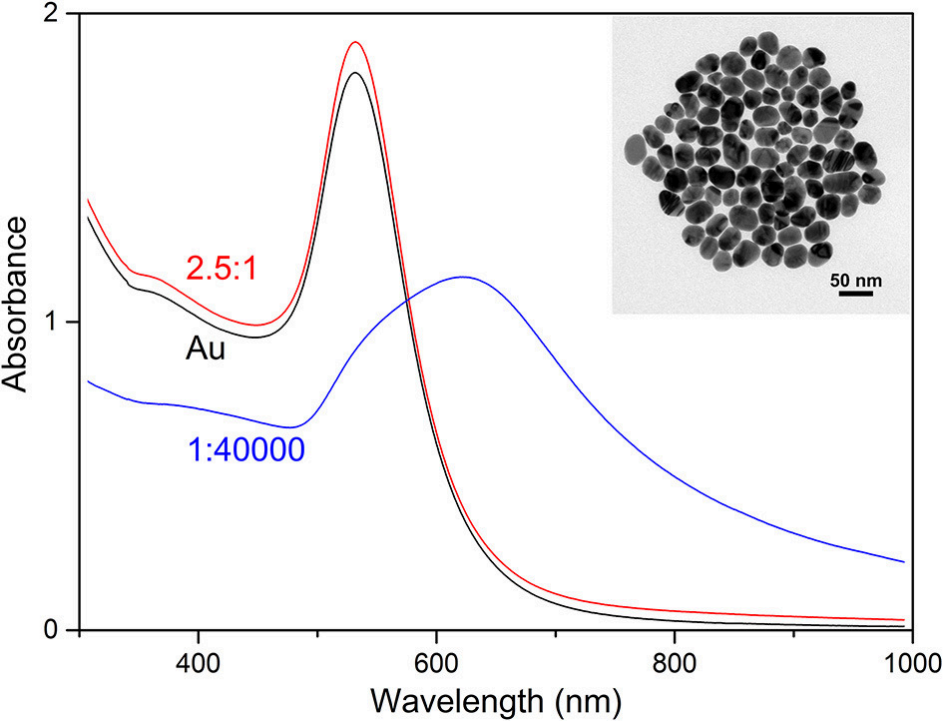
Absorbance spectra of the gold nanoparticles (Au) and the mixture of BSA with the gold nanoparticles at the molar ratios of 2.5:1 and 1:40,000 (nanoparticle: BSA). The inset shows a TEM micrograph of the gold nanoparticles.

Figure 2 shows the UV-vis absorbance spectra of the samples at the nanoparticle: BSA molar ratios of 1:40,000 and 2.5:1, and a spectrum of the nanoparticles without BSA. The results of the sample without BSA show an absorbance maximum at 532 nm, the single, relatively narrow peak suggests relatively high monodispersity of spherical nanoparticles, confirmed by transmission electron microscopy (TEM), which indicates a nanoparticle diameter distribution of 32 ± 7 nm (inset in Figure 2). Upon addition of BSA to the gold nanoparticles to yield a ratio of 40,000 BSA molecules per nanoparticle, aggregates of the nanoparticles form, as indicated by the extension of the plasmon band, with a shift of its maximum to 618 nm. This observation is in accord with data from other albumin-containing samples (Drescher et al., 2013b), where the change in plasmonic properties of these gold nanoparticles was also connected to aggregate formation. Protein molecules are known to aggregate nanoparticles in certain concentration ranges via depletion, bridging, or by reducing the surface charge during adsorption (Bharti et al., 2011; Moerz et al., 2015). In the sample containing gold nanoparticles and BSA molecules at a ratio of 2.5:1, the differences between the spectra are relatively small (compare red and black trace in Figure 2), but the dark field view in the microscope indicates the presence of small gold nanoaggregates as well. This is in accord with the SERS spectra indicating an interaction of the protein with the nanoparticles, and supports a bridging interaction of protein molecules that can connect several individual nanoparticles. As discussed previously, although the formation of such small aggregates has a smaller effect on the bulk absorbance spectrum and can lead to an underestimation of electromagnetic SERS enhancement (Joseph et al., 2011) using a quantitative relation to the absorbance spectra (Weitz et al., 1982), the strong increase in SERS enhancement that is brought about by the formation of such aggregates clearly evidences their presence, albeit often in small numbers, in SERS experiments (Kneipp et al., 1998; Joseph et al., 2011; Madzharova et al., 2018).

**Figure 3:**
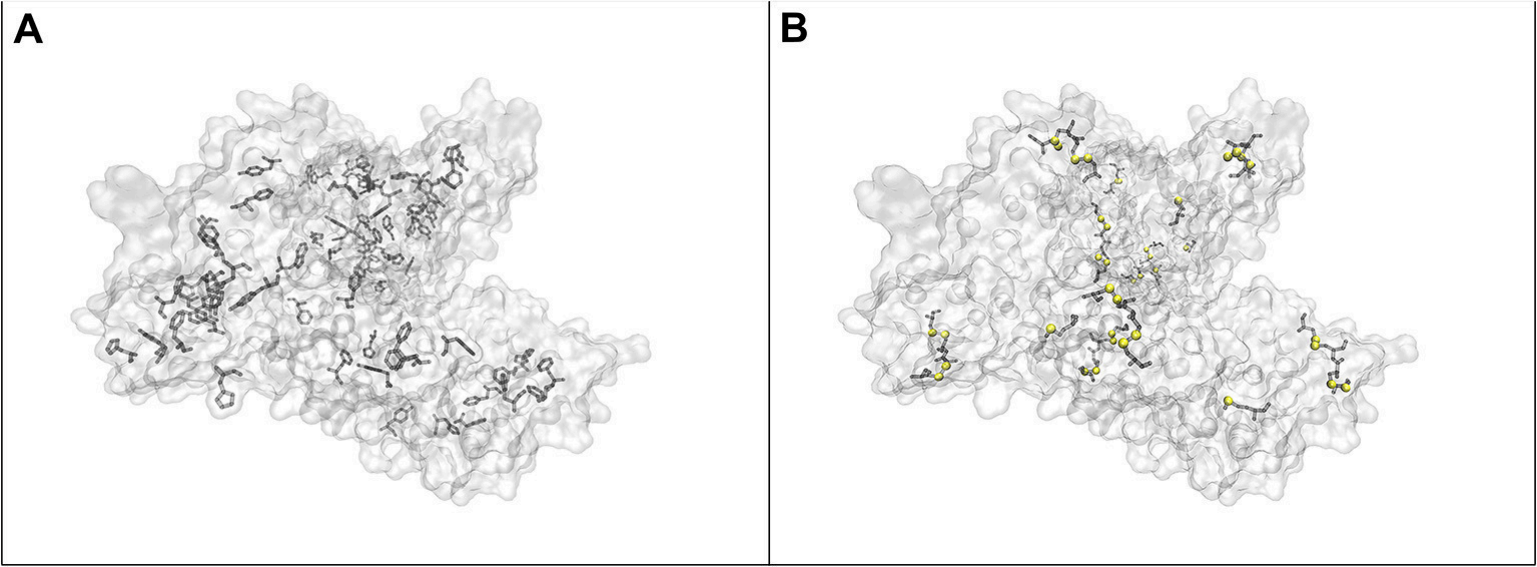
**(A)** Location of the aromatic side chains in BSA, **(B)** location of cysteine and methionine residues in the structure of BSA. (Humphrey et al., 1996; Bujacz et al., 2014) Sulfur atoms are marked with yellow spheres.

Different from well-defined molecular systems and their interaction and coverage of gold surfaces (Liu et al., 2002; Joseph et al., 2011), the number of BSA molecules participating in the SERS enhancement is very hard to estimate in the agglomerates here. As indicated by the overall signals in the spectra in Figure 1, the enhancement by the gold nanoaggregates, whose formation is induced by BSA varies for different protein concentration, in agreement with the concentration–dependent interaction of the protein with the gold nanoparticles (Moerz et al., 2015). This will result in a different portion of nanoparticles present in aggregates, as well as a different aggregate morphology. The latter, specifically the variation of inter-particle distance and the combination of not-ideally spherical nanoparticles, was recently shown to have a strong influence on the local field enhancement of gold nanostructures for excitation in the near-infrared (Madzharova et al., 2018), and is expected to play a major role in the different overall enhancement observed here as well.

In spite of the strong influence of the BSA analyte molecule on the optical properties of the gold nanoparticle aggregates, rendering protein quantification impossible, a comparison of overall SERS signals is interesting regarding the ability to probe protein composition and interaction in cells, where such aggregates are formed from individual gold nanoparticles when they meet with the real biological environment. Comparing the normal Raman signals (Figure 1, black trace) of a 7.5 mM BSA solution with the SERS spectrum obtained at a BSA concentration of ~150 pM (Figure 1, blue trace), we estimate an enhancement of ~10^8^.

It is important to consider that all samples for the SERS experiments (Figure 1) consist of citrate stabilized gold nanoparticles and BSA, and the highest BSA concentration resulted in the lowest signal intensities, rather than in a saturated overall intensity as would be expected due to a limited available surface of the SERS substrate (Joseph et al., 2011). This observation is in agreement with recent discussions (Moerz et al., 2015) that nanoparticle aggregation hardly occurs when the protein concentration is much higher than the concentration needed to form a monolayer on each particle. This is the case in the sample with a gold nanoparticle: BSA molar ratio of 1:10^7^. In a protein-nanoparticle system, the governing transport process of the particles is diffusion by Brownian motion, which depends on the dynamic viscosity η of the system as well. We estimate η of the SERS sample with the gold nanoparticle: BSA ratio of 1:40,000 to be 0.803 cP, while the SERS sample with the gold nanoparticle: BSA ratio of 1:10^7^ exhibits a η of 1.94 cP. Displacement of the nanoparticles also depends on the particle radius that significantly changes upon the adsorption of additional protein layers. We conclude that the slower Brownian motion and the increased minimum inter-particle spacing due to multilayer protein adsorption contributes significantly to the absence of aggregate formation and therefore to the weaker SERS enhancements at higher protein concentrations. Estimating the amount of nanoparticles taken up by a typical cell (Drescher et al., 2012), and the number of typical-size gold nanoaggregates (Drescher et al., 2013b) that would fit in a focal volume, the number of particles is very similar. Since the most concentrated sample approximates the cellular protein concentration, the observed high SERS signals from live cells (Kneipp et al., 2006; Zivanovic et al., 2018) must be related to differences in the intracellular inhomogeneity of protein concentrations and to active transport processes, which facilitate the formation and positioning of aggregates in cellular compartments. Therefore, the combination of these phenomena can contribute to the SERS signal enhancement experienced in live cell SERS mapping.

**Figure 4:**
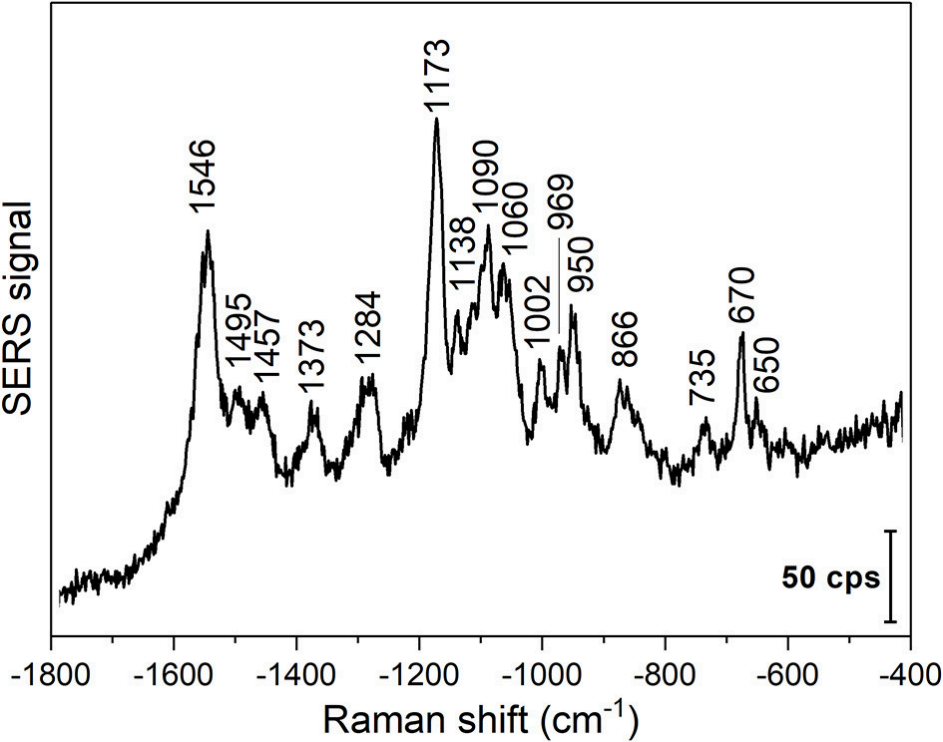
Representative SERS spectrum of 3T3 DNA in the presence of gold nanoparticles. Excitation wavelength: 785 nm, excitation intensity 5.7 × 10^5^ W/cm^2^, acquisition time per spectrum: 1 s.

To show the application of gold nanoparticles and direct SERS probing in other biomolecular solutions, DNA extracted from 3T3 fibroblast cells was used. A representative SERS spectrum of the DNA solution is shown in Figure 4. The SERS experiments were conducted at pH 4.54, which can destabilize the DNA duplexes by titrating the polar groups in the nucleobases (Creighton, 2010). High relative intensities of the vibrations at 1,090 cm^−1^ assigned to ${\text{PO}}_{2}^{-}$ symmetric oxygen stretching (Peticolas, 1995; Huser et al., 2009), at 1,173 cm^−1^, corresponding to base external C-N vibrations (Small and Peticolas, 1971), and at 1,546 cm^−1^ from NH_2_ scissoring in adenine, N3-C4-C5 stretching in cytosine, and C-N stretching, NH_2_ scissoring, and N1-H bending in guanine (Madzharova et al., 2016) are observed. The lower relative intensity of the deoxyribonucleoside and backbone vibrations (Figure 4 and Table 3), and the appearance of the 797 cm^−1^ band in the spectra indicate the destabilization of the double helix (Small and Peticolas, 1971), as well as the proximity and possible interaction of the nucleobases and the gold nanoparticle surface. Due to the short experiment times, we can exclude depurination in the DNA strands that was reported to occur at low pH (An et al., 2014). The spectrum obtained with purified DNA suggests that biomolecule-nanoparticle interactions other than those of proteins can be studied, and that structural information about the state of the biomolecules can be obtained.

**Table 3:** Raman shift values and tentative band assignments of the SERS spectrum of 3T3 DNA.

**Raman shift (cm^**−1**^)**	**Tentative assignment**
1,546	A, C, G
1,495	A
1,457	A, G, T
1,373	dA, dC, dT
1,284	T
1,173	Base external C-N stretch
1,138	A
1,090	ν(${\text{PO}}_{2}^{-}$)
1,060	G
1,002	A, C
969	A, G
950	G
866	G
797	Sugar-phosphate symmetric stretch
735	A
670	dT
650	dC, backbone

*Tentative band assignments are based on Small and Peticolas (1971), Erfurth et al. (1975), Peticolas (1995), Huser et al. (2009), and Madzharova et al. (2016)*.

In conclusion, we have presented here a sample preparation approach for obtaining SERS spectra of biomolecules in a setting that resembles the application of individual, non-aggregated gold nanoparticles in a complex biosystem such as a live cell. SERS spectra were measured in solutions of the protein BSA over a wide range of concentrations and in the absence of aggregating agents. Aggregates of the gold nanoparticles are formed by interaction with the protein molecules. As indicated by the SERS spectra, the BSA molecules undergo a change in structure, which depends on their concentration. Specifically at low gold nanoparticle: protein molar ratio, significant unfolding of the protein is observed, in agreement with the expected aggregation mechanism at low concentrations. Apart from qualitative changes in the spectra, the varied interaction of the proteins with the gold nanoparticles modifies aggregate morphology, yielding a great variation in SERS enhancement. When protein concentrations in the solutions approximate cellular protein concentrations, the high viscosity and the multilayer protein adsorption on the surface of the nanoparticles hinder the formation of nanoaggregates that can provide high SERS enhancement. These results show that the high SERS signals in live cells are the consequence of additional positioning and formation of intracellular aggregates apart from the crowded environment inside a cell alone.

## Author Contributions

GS and JK contributed equally. GS participated in the experimental planning, experimental execution, and the writing of the manuscript. JK participated in the experimental planning and the writing of the manuscript.

### Conflict of Interest Statement

The authors declare that the research was conducted in the absence of any commercial or financial relationships that could be construed as a potential conflict of interest.
